# The Impact of Racially Motivated Housing Discrimination on Allostatic Load among Indigenous University Students

**DOI:** 10.1007/s11524-020-00446-6

**Published:** 2020-06-03

**Authors:** Cheryl L. Currie, Takara Motz, Jennifer L. Copeland

**Affiliations:** 1 Faculty of Health Sciences, University of Lethbridge, M3083 Markin Hall, 4401 University Drive, Lethbridge, AB T1K3M4 Canada; 2 Department of Kinesiology and Physical Education, Faculty of Arts & Science, University of Lethbridge, 4401 University Drive, Lethbridge, AB T1K3M4 Canada

**Keywords:** Racial discrimination, Housing discrimination, Allostatic load, Indigenous

## Abstract

Allostatic load (AL) is an aggregate measure of wear and tear on the body due to the chronic activation of the stress response system. The goal of this study was to examine the association between racially motivated housing discrimination (HD) and AL score within a sample of Indigenous university students. Data for this cross-sectional study were collected from Indigenous adults attending university in a small city in western Canada between 2015 and 2017 (*N* = 104; mean age = 27.8 years). An item adapted from the Experience of Discrimination Scale was to assess racially motivated HD in the past 12 months. AL was measured as a composite of 7 biomarkers assessing neuroendocrine, cardiovascular, metabolic, and immune system function. Bias-corrected and accelerated bootstrapped linear regression models were used to examine associations adjusting for age, income, parenthood, and other situations in which discrimination had been experienced. Indigenous university students who experienced racially motivated HD in the past year (16.8% of the sample) had an average AL score of approximately 4, which was almost double that of their peers who had not. In an adjusted model, racially motivated HD was associated with a 1.5 point increase in AL score. This model explained 35% of the adjusted variance in AL score, of which racially motivated HD explained 24%. These results suggest Indigenous adults who experienced racially motivated HD in the past year had early and more pronounced wear and tear on neuroendocrine, cardiovascular, metabolic, and immune system functioning in young and middle adulthood than Indigenous peers who did not. These findings combine with others to highlight the need for increased efforts to prevent racially motivated HD in urban centers.

## Background

Racial discrimination is widely recognized as a fundamental social determinant of health for racialized groups [1]. There has been limited information about the impacts that racially motivated housing discrimination (HD), defined as HD motivated by race, ethnicity, or color, has on health. This examination is important given housing is an essential human resource and key determinant of health. Limiting access to housing can directly endanger the physical welfare of individuals by pushing them into substandard or unsafe living conditions, secondary homelessness (transient or emergency accommodation), and primary homelessness (no accommodation) in cities.

Racially motivated HD is commonly experienced by minority populations. In the USA, audits measuring HD across 5500 paired tests in 30 urban areas found African Americans and Hispanics experienced consistent adverse discriminatory treatment in approximately 20–25% of housing rental or sales searches [2]. For renters, this included receiving less information and fewer opportunities to view units, while home buyers received less assistance with financing and were steered into less wealthy communities by real estate agents. A study that examined 369 paired tests in Sydney, Australia, found rental agents treated prospective renters originating from India and the Middle East differently from Anglo Australians, including providing them with less information and follow-up [3]. A sample of Indigenous adults described the search for housing in Sydney as a battle and a pervasive source of stress affecting the health of the whole Indigenous community living there [4]. While social networks were used to avoid primary homelessness, discrimination directed at Indigenous people was contributing to long wait lists for social housing [4]. In Canada, studies across three cities suggest approximately a third of Indigenous adults experienced adverse discriminatory treatment in housing rental and sales searches [5–7]. For renters, this included fewer opportunities to view units, being denied a rental application because of being Indigenous, and being told places were “just rented” when they had not been. For home buyers, this included differential treatment from real estate agents and mortgage agencies [5].

Studies published by Yang and colleagues using data collected from approximately 10,000 adults living in 830 US neighborhoods found racially motivated HD was associated with reduced self-reported health, increased self-reported stress, and higher chronic disease and mental health disorder diagnoses [8–10]. Several qualitative and mixed methods studies have similarly reported impacts on mental and physical health [4, 11, 12]. Nightingale (2016) interviewed post-secondary students in Canada about racial discrimination experienced while searching for or maintaining off-campus housing [11]. Participants described how they avoided searching for housing in areas of the city that were “not ethnic friendly” even when those locations were closer to their school [11]. Forms of discrimination documented were similar to those of other studies despite the expanded scope of ethnicities examined [5, 11, 12]. Motz et al. (2019) used the same dataset examined in the present study and found racially motivated HD in the past year was associated with increased PTSD symptomology among Indigenous university students, particularly intrusive recollection [12]. Participants across several studies also described the discrimination they faced *after* they were housed, such as receiving less maintenance services than other tenants, feeling pressured to move, and the stress and hopelessness they felt over this ongoing abuse of power [4, 5, 11].

We theorize that the symptoms of mental distress reported by participants who experience racially motivated HD may go beyond the mind to impact *stress biology*, thus serving as a pathway through which HD may get under the skin to impact the health outcomes reported by Yang and colleagues [8–10]. The human homeostatic system responds to a wide variety of environmental and social stressors by producing hormonal and neurotransmitter mediators that set appropriate physiologic responses in motion [13]. Yet some stressors, particularly those that threaten survival by creating uncertainty about access to essential human resources like housing, may perturb the body in ways that result in a shift from a homeostatic to an *allostatic state* [14–16]. An allostatic state, sometimes called fight-or-flight, is an altered form of physiologic regulation designed to promote short-term survival in the face of significant threat to personal safety [13]. Allostasis involves overresponses across one or more biological markers in magnitude or duration, as well as multiple physiologic responses competing concurrently with one another in an effort to promote short-term survival [16, 17], while adaptive in the short-term chronic overresponding can lead to changes in the body’s defended values, range, or diurnal pattern for an array of biomarkers (e.g., cortisol). Also, multiple physiologic responses competing concurrently with one another is inefficient in the longer term and can lead to the weathering of these systems [16, 18, 19]. AL is an aggregate measure of wear and tear on the body due to the chronic activation of allostasis [20]. Increased AL is a preclinical marker of the pathophysiologic processes that precede the onset of disease, and a 10-year predictor of morbidity, all-cause mortality, and cause-specific mortality across young, middle-aged, and older age adults [21, 22].

We posit that racially motivated HD may serve as a triggering experience that could shift the body into chronic allostasis and thus increased AL, given HD threatens access to an essential human resource by interfering with the ability to achieve safe, stable, and appropriate shelter. To test this hypothesis, we examined whether racially motivated HD experienced in the past 12 months was associated with increased AL in a relatively young sample (mean age = 27.8 years) of Indigenous adults attending university in a small urban center. AL was operationalized using markers from the three biological domains that framed the original AL index (i.e., neuroendocrine, cardiovascular, metabolic), and an added immune marker [23].

### Population Focus

Approximately 5% of Canadians identify as Indigenous [24]. The three main Indigenous groups in Canada—First Nations, Métis, and Inuit—together represent a diverse range of histories, cultures, and languages [24]. A majority of Indigenous Canadians (58%) identify as First Nations, including those who have Treaty Indian Status under the Indian Act and those who do not. More than a third (35%) of Indigenous people identify as Métis, who are broadly defined as individuals with mixed First Nations and European ancestry (particularly French) [24, 25]. Inuit are the original people of the North American Arctic and make up 4% of those who identify as Indigenous in Canada. The large majority of this population continues to live in Inuit Nunangat, which is the homeland of Inuit peoples in northern Canada [24, 26].

The average age of the Indigenous population (32 years) is almost a decade younger than that of non-Indigenous Canadians (41 years). More than half of all Indigenous Canadians aged 25–64 years have a post-secondary qualification, including 34% who have completed university or college [27]. More than one in two (54%) Indigenous Canadians live in a city [24]. The proportion of the urban Indigenous population living in low-income households declined from 28% in 2006 to 24% in 2016, but remains higher than the non-Indigenous population [28]. Approximately half of all Indigenous Canadians living in cities rent their dwelling compared with one-quarter of the non-Indigenous population [28].

Various studies suggest Indigenous populations experience high levels of racial discrimination relative to other Canadians [6, 29–31]. Nationally, the most recent estimate suggests 20% of all Canadians report discrimination due to their race at least sometimes, compared with 73% of Canadians who identify as Indigenous [32]. When asked to identify, without prompting, the racial groups who most frequently experience discrimination in Canada, 23% of the general population identified Indigenous people, followed by 16% who identified Africans or Blacks [32].

## Methods

The present analysis is based on a cross-sectional study that collected data on the social determinants of Indigenous health among 150 university students who identified as Indigenous and attended school in a small city in western Canada (population size 100,000). Data collection began in September 2015 and continued over 4 academic terms ending in April 2017. Study procedures were approved by the Human Subjects Research Committee at the University of Lethbridge. Data analyzed for the present analysis are available from the corresponding author upon reasonable request.

### Indigenous Advisory Committee

This study was conceptualized using a participatory action research framework [33]. An Indigenous Advisory Committee made up of key members of Indigenous organizations in the city in which this project took place was assembled and worked with the research team to set study priorities and make data collection decisions. Working together with this Committee, we determined that salivary samples would examine AL, and that a system would be put in place to respect the wishes of Indigenous participants in relation to these samples. As described by the Tri Council Policy Statement 2 (Article 9.8), researchers have an obligation to become informed about, and to respect, relevant customs and codes of practice that may apply in a particular territory [34]. Article 9.19 notes that researchers should be aware that Indigenous people may seek to maintain control over human biological materials collected for research, in accordance with Indigenous world views about “full embodiment” in which all parts and products of the body are sacred [34]. Thus, our consent form provided participants the option of having their saliva samples returned to them upon analysis, or to have their saliva samples included in an Indigenous ceremony led by an Indigenous Knowledge Holder that returned the samples to the Earth.

### Sample and Procedures

Posters and ads placed in e-newsletters were used to recruit volunteers from among the 400 Indigenous students attending the university during the data collection period. Respondents were asked to confirm eligibility by email/phone (i.e., they identified as Indigenous, current post-secondary students, and 18 years or older). Participants then attended an on-campus study office to complete consent procedures, paper-and pencil surveys, and the physical assessments needed to calculate AL score (mean completion time = 90 min) during standard office hours (9:00 am–4:00 pm). To ensure sufficient recruitment, we needed to accommodate student course schedules and thus could not standardize a narrow window for data collection, which may have been useful for some biomarkers examined (e.g., DHEA-S, CRP). Saliva samples were collected at 3 time points during the office visit using the passive drool technique. Participants rinsed their mouth with water and the first sample was collected after completing a portion of the questionnaire. Remaining samples were taken 30 and 60 min later. Whole saliva samples were collected in a 2-ml microcentrifuge tube using a Saliva Collection Aid (Salimetrics, State College, PA). During data collection, salivary samples were stored in the in-office freezer and then transferred to a − 80 °C freezer. Participants were provided with supplies for collecting saliva samples at home for 2 days, and contact information for the research assistant working with them.

At home, participants selected two consecutive days with similar wake/sleep times and collected a saliva sample at three time points: immediately upon wake-up, 30 min after wake-up, and before bed, and to record the times in which samples were taken on forms provided. Participants were instructed to place the swab under the tongue for 3 min, and then place it in a pre-labeled tube and put it in their freezer. When all six samples were collected, the participant contacted the research assistant to coordinate sample return. We used cortisol awakening response (CAR) expert consensus guidelines to increase at-home adherence including clearly explaining the importance of strict adherence to sampling times, emphasizing the importance of collecting sample S1 immediately upon awakening, encouraging participants to ask questions via text/email/phone, providing take-home instructions, having participants record data collection time points in a diary log, advising participants to place kits beside the bed for morning collection, and text messaging the evening before sampling to highlight instructions [35]. Participants returned the samples in an insulated lunch kit with a freezer pack given to them during the in-office visit. Samples received were transferred to a − 80 °C freezer. Participants were given an honorarium of $50 for in-office measures and $50 for at-home measures.

### Measures

#### Allostatic Load

AL score was based on a composite of seven biomarkers across four biological domains:

1. Cardiovascular markers: Resting systolic and diastolic blood pressure were measured using a Life Source automated sphygmomanometer (Auto Control Medical, Mississauga, ON). The first was taken approximately 15 min after the participant arrived, once they had completed the consent process and answered the first part of the survey package in a seated position. This reading was discarded. Two additional readings were taken 15 and 30 min after the first while the participant was seated. These two measures were averaged.
2. Neuroendocrine markers included DHEA-S and CAR. All were analyzed in duplicate. As per manufacturer’s suggestion for DHEA-S, the three in-office samples were pooled and mixed for analysis. To examine CAR, the wake-up (S1) and 30 min post wake-up (S2) samples taken at home on the second day were used to calculate the percent change in cortisol between S1 and S2. Day 1 at-home samples were not combined with day 2 to produce an average because missing data were higher on day 1. CAR represents the sharp rise in cortisol levels across the first 30–45 min following morning awakening. In healthy adults, the magnitude of CAR ranges between 50 and 156% [36]. The mean CAR magnitude in this study was 65.1% (Table 1).
3. Metabolic markers included body mass index (BMI) and waist circumference. To calculate BMI, height and weight were measured to the nearest 0.5 cm using a Health O Meter mechanical beam scale and stadiometer, and to the nearly 0.1 kg using a weighbeam scale, respectively. Waist circumference (WC) was measured at the top of the iliac crest to the nearest 0.5 cm. Although correlated (Pearson’s *r* = 0.87 in this sample), both measures were included in the AL score as each is independently associated with health risk.
4. Immune marker: We measured CRP using the third in-office saliva sample.

**Table 1:** Mean, range and cut-points used for allostatic load (AL) biomarkers (*N* = 104)

Biomarker	Range	Mean	SD	Cut-point female	Cut-point male
1. Cardiovascular					
Resting SBP (mm Hg)	90, 150	119.1	13.0	> 140	> 140
Resting DBP (mm Hg)	59, 111	78.0	10.3	> 90	> 90
2. Neuroendocrine					
DHEA-S (μg/dl)	188.5, 16,055.6	4247.0	3743.0	< 1419.5	< 2865.1
CAR	− 98.8, 771.7	65.1	165.4	< 50.0 or > 156.0	< 50.0 or 156.0
3. Metabolic					
BMI (kg/m^2^)	18.8, 48.5	29.0	6.5	> 30.0	> 30.0
Waist circumference (cm)	68.9, 166.4	97.9	18.1	> 88.0	> 102.0
4. Immune					
C-Reactive protein (pg/ml)	55.1, 3150.0	481.9	666.9	> 397.8	> 711.8
Total AL score	0–6	2.5	1.3		

*SBP*, systolic blood pressure; *DBP*, diastolic blood pressure; *DHEA-S*, dehydroepiandrosterone-sulfate; *CAR*, cortisol awakening response; *BMI*, body mass index

Cortisol, DHEA-S, and CRP concentrations were assessed using enzyme-linked immunosorbent assays (ELISA) (Salimetrics, LLC., State College, PA). Average intra-assay variability was 3.9% for cortisol, 6.6% for DHEAS, and 4.3% for CRP. Average inter-assay variability was 9.2% for cortisol, 12.8% for DHEA-S, and 8.3% for CRP. For CAR, all samples from the same participant were analyzed in the same plate, to minimize the effect of inter-assay variability. AL risk assessment was based on the distribution of the study sample for salivary CRP and DHEA by dividing the sample into sex-specific quartiles with high risk defined by the highest quartile for CRP and the lowest quartile for DHEA-S. As shown in Table 1, we used standard cutoffs for all other biomarkers [37, 38]. Consistent with prior studies, one point was assigned if the variable was in the high-risk quartile and 0 if not. Scores were summed across each system type (neuroendocrine, metabolic, immune, and cardiovascular) to create a total score for AL.

#### Racial Discrimination

Racially motivated HD was operationalized using an adapted question from the Experiences of Discrimination (EOD) Scale: “In the past 12 months, have you experienced discrimination, or been hassled or made to feel inferior getting or maintaining housing because of your Aboriginal race, ethnicity, or color?” Response options were 0 = No and 1 *=* Yes [39]. We chose to operationalize HD in this way for comparability with the three quantitative publications that have examined the impact of HD on health in the literature to date [9, 10, 40].

#### Covariates

Exact age, gender, parenthood, and income were collected as part of the survey package. Categories used to examine each covariate are outlined in Table 2.

**Table 2:** Characteristics of the sample

Characteristics	Total *N* (%)
Total sample	104 (100%)
Gender	
Female	76 (73.1)
Male	28 (26.9)
Age	
18–24 years	46 (44.2)
25–34 years	36 (34.6)
35–44 years	17 (16.3)
45+ years	5 (4.8)
University student status	
Full-time student	102 (98.1)
Part-time student	2 (1.9)
Rent or own home	
Rent	75 (72.1)
Own	29 (27.9)
Where do you live	
On campus	5 (4.8)
Off campus	99 (95.2)
Currently employed	
Yes	25 (27.9)
No	75 (72.1)
Income group	
Upper-middle/upper income	7 (6.7)
Upper-middle/middle	25 (24.0)
Lower-middle	51 (49.0)
Low income	21 (20.2)
Marital status	
Single	64 (61.5)
Married/living common-law	40 (38.5)
Have children (parenthood)	
Yes	45 (43.3)
No	59 (56.7)
Past year housing discrimination	
Yes	17 (16.3)
No	87 (83.7)
Mean allostatic load score (SD, range)	
Housing discrimination: yes	3.9 (1.1, 2–5)
Housing discrimination: no	2.2 (1.1, 0–6)

### Missing Data

Data were collected from 150 participants: 35 of whom were removed from the analysis because they chose to not complete and/or return at-home samples. An additional 8 were removed because the timing of at-home sampling was completed in ways that did not follow procedure resulting in the inability to calculate valid CAR [35]. Also, two participants were removed for not completing questions about discrimination in the past 12 months, and one was removed for not reporting their age. There were no missing data on survey questions about gender or income. The final sample size included in this analysis was *N* = 104. Independent-samples *t* tests confirmed the mean age, income, and HD experience of participants included and excluded from the analysis due to missing data were not statistically different, nor was the gender balance different between groups. We conducted a supplementary reanalysis of the main findings excluding CAR from the AL calculation, which reduced our ability to understand the impacts of HD on neuroendocrine function, but increased the sample size to *N* = 144.

### Analysis Strategy

Bootstrapped linear regression models (*k* = 5000) examined the association between past-year HD (yes or no) and the continuous form of AL. Bias-corrected and accelerated (BCa) bootstrap intervals were used to adjust for potential skew. Potential confounders were carefully considered and tested before inclusion in models to reduce model overfitting, and keeping in mind that analyses that follow the “more control variables is better” approach to improve causal inference have been debunked [41–44]. Thus, potential confounders were tested using individual regression models before entry into the main model. Those associated with AL at *p* < 0.10 were retained [45] which included age, income, and parenthood. All analyses were run using SPSS 25.0.

A sample size calculation could not be estimated given the associations examined were novel for the population under study, and there were a dearth of studies within other populations that could be used to estimate sample size when this study began in 2015. We have conducted a post hoc power calculation for readers who may be interested, keeping in mind the cautions put forward about doing so [46–48]. A *t* test was used given the exposure was dichotomous and the outcome continuous. The mean AL score in persons who had, and had not, experienced HD in the past year was 3.9 (SD 1.1) and 2.2 (SD 1.1), respectively, a 73% increase. To detect a 70% increase or more in mean AL score between two groups, 34 participants per group is recommended to achieve 80% power for this effect size using a two-tailed test [49]. If it could be assumed that the observed effect size in our study was similar to the true effect size in the population, then our full model was somewhat underpowered to detect statistical significance between groups, given 17 participants were exposed to HD in the past year and 87 participants not.

## Results

### Sample Characteristics

Sample characteristics are shown in Table 2. All participants were adult post-secondary students and most (98.1%) attended school full-time. Almost three-quarters of the sample were female in keeping with higher proportions of female Indigenous students in Canadian universities [50]. The mean age was 27.8 years (SD = 8.7, range 18–57 years), which is somewhat lower than the mean age (32.1 years) of Indigenous people in Canada during this time frame [24]. Approximately 4 in 10 students were married or living with partner, and 43.3% had children. Most (69.2%) identified as low–middle or low income, and approximately 3 in 10 students were employed. As shown in Table 1, most students were living in rental housing off campus.

The mean AL score for this sample was 2.5 out of a possible 7 (SD 1.3, range 0 to 6). The AL median and mode were both 2.0, and the AL skewness value was 0.5 suggesting the distribution of AL scores were approximately symmetric.

Overall, 16.3% of the sample had experienced racially motivated discrimination while searching for or maintaining housing in the past 12 months. No students living on campus reported HD. Students between the ages of 25–44 years, and those who were parents were significantly more likely to experience HD than other participants (Chi-square = 12.1, df = 3, *p* = 0.007; and 12.7, df = 1, *p* = 0.001; respectively).

### Racially Motivated HD and AL

As shown in Table 1, the average AL score was 3.94 (SD = 1.14) among adults who experienced HD, which was almost double that of those who had not had this experience in the past year (mean AL = 2.22, SD = 1.14, independent-samples *t* = 5.71, df = 103, *p* < 0.001). In a model adjusted for sociodemographics, adults who experienced racially motivated HD in the past year had AL scores that averaged 1.5 points higher than peers who had not (Table 3, model 2). The full model explained 36% of the variance in AL score, of which racially motivated HD explained 24% (*R*^2^ Change = 0.24, *F* Change = 32.52, Sig. *F* Change *p* < 0.001).

**Table 3:** Linear regression models for the direct effects of racially motivated housing discrimination (HD) on AL score calculated with CAR (models 1–3, *N* = 104) and without CAR (models 4–6, *N* = 142)

Model	Adj *R*^2^	*β*	SE	*B* (95% CI)	*p*
AL with CAR					
1	0.23	0.49	0.30	1.73 (1.13, 2.32)	0.001
2	0.36	0.44	0.29	1.51 (0.93, 2.09)	0.001
3	0.35	0.44	0.32	1.50 (0.87, 2.14)	0.001
AL without CAR					
4	0.10	0.32	0.28	1.15 (0.60, 1.71)	0.001
5	0.26	0.26	0.27	0.94 (0.40, 1.49)	0.001
6	0.26	0.22	0.30	0.80 (0.20, 1.40)	0.01

*β*, standardized beta weight; *B*, unstandardized beta weight; *SE*, standard error; *AL*, allostatic load; *CAR*, cortisol awakening response. *Models 1, 4* unadjusted estimates; *Models 2, 5* estimates adjusted for age, income, and parenthood; *Models 3, 6* estimates adjusted for age, income, parenthood and other forms of racial discrimination experienced in the past year

The impact of HD on AL may be due to heightened discrimination experienced in other situations in that time frame, given those who reported HD in the past year experienced discrimination across significantly more situations in the past year than those who had not (mean = 5.1 vs. 1.8 situations, independent-samples *t* = 7.44, df = 101, *p* < 0.001). To examine this, we computed the total number of situations discrimination had been experienced in the past year, excluding housing, and added this covariate to the model. The variance inflation factor (VIF) was 1.3 indicating that the variance of this new covariate was 1.3 times greater than it would be if it was linearly independent of the other variables in the analysis [51]. This is below generally recommended thresholds and suggests multicollinearity was not a significant concern [52]. Findings indicate no change in the strength or significance of the association between HD and AL when discrimination experienced in other situations in the past year was controlled (Table 3, model 3).

### Supplementary Analyses

Table 3 outlines a reanalysis of the main findings excluding CAR from the AL calculation. This decreased our understanding of the impacts of HD on neuroendocrine function but increased the sample size to *N* = 144. The mean AL score when calculated without CAR was 1.8 out of a possible 6 (SD 1.3, range 0 to 5). Among those who experienced HD in the past year, the average AL score, excluding CAR, was 2.77 (SD = 1.23), compared with 1.62 among those who did not (SD = 1.22, independent-samples *t* = 4.06, df = 144, *p* = 0.001). Those who experienced HD had AL scores that were almost one point higher than those of their peers who had not; this difference remained statistically significant when sociodemographic confounders and other forms of discrimination in the past year were controlled for (Table 3, models 5–6). The full model explained 26% of the variance in AL score, of which racially motivated HD explained 10% (*R*^2^ Change = 0.10, *F* Change = 16.10, Sig. *F* Change *p* < 0.001).

## Discussion

Racially motivated HD was a risk factor associated with a 1.5 point increase in allostatic load (out of a possible 7 points) within a relatively young sample of Indigenous university students in Canada. The aggregate measure of AL used in this study suggests Indigenous adults who experienced racially motivated HD had early and more pronounced wear and tear on neuroendocrine, cardiovascular, metabolic, and immune system functioning in young and middle adulthood than peers who did not. These findings corroborate previous research documenting an association between racial discrimination experienced across a variety of life situations and AL [45, 53–55]. We add to this literature by demonstrating that a *setting-specific* form of racial discrimination—racially motivated HD—was independently associated with AL score after accounting for other situations in which discrimination had been experienced in the same time period. Given AL is a preclinical marker of the disease process, increased AL observed among those who experienced HD in this study may serve as an etiologic pathway linking HD to the adverse health outcomes described in the literature [4, 5, 8–11, 20–22].

Several explanations for the observed association between racially motivated HD and AL are possible. Housing discrimination may have been perceived, consciously or unconsciously, as a significant threat to physical safety by participants, given housing is an essential resource for human survival, and HD creates uncertainty about whether one can secure and retain this resource for themselves and their family. Thus, HD may have perturbed the stress response system toward short-term survival and an allostatic state. This would explain higher AL among adults who experienced HD in this study, given AL is an aggregate measure of physiologic wear and tear due to the chronic activation of allostasis. It may also be posited that when motivated by race, HD becomes exceptionally threatening, given it is based on fixed features of an individual’s physical appearance that cannot be easily modified. Thus, HD due to race would make it more difficult for an individual to control or moderate the situation and gain access to this resource.

Due to the cross-sectional nature of the data used in the present study, the hypothesized temporal sequence of the observed association between racially motivated HD and AL remains tentative without further research. It is possible that renters with higher AL scores appeared different in ways that made them less desirable tenants. Yet, it is difficult to establish how individuals screening tenants for units would ascertain or be aware of AL scores in ways that would be consistent enough to produce the observed associations. There is stronger biological plausibility for the argument that racially motivated HD preceded increased AL. Also, longitudinal research has linked adolescent experiences of racial discrimination between 16 and 18 years of age to elevated AL at age 20, thus suggesting a temporal sequence for these variables among young adults [55].

### Ways Forward

The Canadian Truth and Reconciliation Commission (TRC), struck in 2009, interviewed more than 7000 residential school survivors and their families [56]. The 2015 TRC final report highlights *education* as the key to reconciling the troubled relations between Indigenous and non-Indigenous Canadians [57]. The TRC (2015) has called upon all levels of government to provide education to public servants—medical, nursing, and law students—and staff working within corporations across the country to remedy the gaps in historical and current knowledge that perpetuate ignorance about Indigenous peoples [58]. As noted within the TRC final report, this education must also include anti-racism and cultural competency training to counter the dehumanization of Indigenous people in historical and literary writings, which continue to shape beliefs about Indigenous peoples, particularly in urban centers [58, 59]. The present findings also highlight the need for anti-racism training and monitoring within housing rental systems to ensure Indigenous university students attending school in cities have the same access to housing as their non-Indigenous peers.

### Strengths and Limitations

To date, few published studies have focused on the health impacts of *setting-specific* forms of racial discrimination; this study adds to much needed work in this area. Other strengths include guidance by an Indigenous Advisory Committee, the use of a validated measure of racial discrimination, and the use of a cumulative AL approach to examine the impact of HD on multisystem biological dysfunction. Limiting participants to post-secondary students provided a more homogeneous sample and reduced residual confounding due to educational attainment. Previous studies have controlled for health-risk behavior and mental health when examining associations between discrimination and AL. We disagree with such an approach given racial discrimination predicts later mental health and health-risk behavior problems in more than 30 longitudinal studies, and thus should be examined as part of the causal chain linking various forms of racial discrimination to AL (i.e., as mediators), rather than noise that is controlled in statistical models [1, 60, 61]. Another strength is a sensitivity analysis of the main findings excluding CAR from the AL calculation, which decreased our understanding of the impacts of HD on neuroendocrine function but increased the sample size to *N* = 144. Racially motivated HD remained significantly associated with increased AL in this larger sample when cortisol was excluded.

Limitations include use of a cross-sectional design which precludes inferences about causation and temporal sequence, more female than male participants, and a relatively small sample of participants that may not be generalizable to the general population. The frequency of HD was likely an underestimate as students were not asked if they still lived with their parents. Approximately 30% of the sample was excluded from the analysis due to missing data. However, comparative analyses suggest those excluded did not differ on variables examined, and racially motivated HD remained a significant predictor of AL when a modified version that involved the larger sample was used. Response bias due to self-report is a concern given discrimination is often implicit in nature and pervasive in society and may not always be consciously perceived [39]. Thus, self-reported discrimination may result in underreporting, revealing only a small portion of the actual effect of racial discrimination on the individual [62]. The use of a 12-month measure of discrimination may be a limitation as the heightened AL scores observed may have been a result of HD experienced over a longer time period.

### Conclusion

Indigenous university students who experienced racially motivated HD had early and more pronounced wear and tear on neuroendocrine, cardiovascular, metabolic, and immune system functioning in young and middle adulthood than peers who did not. These findings combine with others to highlight the need for increased efforts to prevent racially motivated HD.

## Data Availability

The datasets analyzed during the current study are available from the corresponding author on reasonable request.

## Abbreviations

AL: Allostatic load
BCa: Bias-corrected and accelerated
BMI: Body mass index
CAR: Cortisol awakening response
CC: Cultural continuity
CRP: C-Reactive protein
DHEA-S: Dehydroepiandrosterone sulfate
EOD: Experiences of discrimination
DBP: Diastolic blood pressure
HD: Housing discrimination
HPA: Hypothalamic-pituitary-adrenal
LOWESS: Locally weighted scatterplot smoother
SAM: Sympathetic-adrenal-medullary
SBP: Systolic blood pressure
VI: Vancouver Index
VIF: Variance inflation factor
WI: Waist circumference
